# Exploring the intrinsic differences among breast tumor subtypes defined using immunohistochemistry markers based on the decision tree

**DOI:** 10.1038/srep35773

**Published:** 2016-10-27

**Authors:** Yang Li, Xu-Qing Tang, Zhonghu Bai, Xiaofeng Dai

**Affiliations:** 1 School of Science, Jiangnan University, Wuxi 214122, China; 2 National Engineering Laboratory for Cereal Fermentation Technology, Jiangnan University, Wuxi 214122, China; 3 School of Biotechnology, Jiangnan University, Wuxi 214122, China

## Abstract

Exploring the intrinsic differences among breast cancer subtypes is of crucial importance for precise diagnosis and therapeutic decision-making in diseases of high heterogeneity. The subtypes defined with several layers of information are related but not consistent, especially using immunohistochemistry markers and gene expression profiling. Here, we explored the intrinsic differences among the subtypes defined by the estrogen receptor, progesterone receptor and human epidermal growth factor receptor 2 based on the decision tree. We identified 30 mRNAs and 7 miRNAs differentially expressed along the tree’s branches. The final signature panel contained 30 mRNAs, whose performance was validated using two public datasets based on 3 well-known classifiers. The network and pathway analysis were explored for feature genes, from which key molecules including FOXQ1 and SFRP1 were revealed to be densely connected with other molecules and participate in the validated metabolic pathways. Our study uncovered the differences among the four IHC-defined breast tumor subtypes at the mRNA and miRNA levels, presented a novel signature for breast tumor subtyping, and identified several key molecules potentially driving the heterogeneity of such tumors. The results help us further understand breast tumor heterogeneity, which could be availed in clinics.

Breast cancer (BC) covers a group of heterogeneous diseases with different biologic, clinical, and molecular characteristics^1^,^2^,^3^. It is important to classify breast cancers into clinically relevant subtypes for therapeutic decision-making and prognosis prediction^4^,^5^. Classically, several different subtypes have been defined using immunohistochemistry (IHC) markers together with clinicopathologic indexes. IHC molecules, containing estrogen receptor (ER), progesterone receptor (PR) and human epidermal growth factor receptor 2 (HER2), have been traditionally used to classify breast tumors^6^,^7^. Notably, PR status is of high correlation with that of ER, leaving ER and HER2 the determinant factors for endocrine and trastuzumab therapy. ER positive and negative tumors have distinctive clinical features and behaviors^7^,^8^. Furthermore, HER2 is the member of the epidermal growth factor receptor family, which is well applied for prognosis and used for sub-classifying ER+ or ER– tumors into distinct subgroups, i.e., [ER+|PR+]HER2− (positive ER and PR status, and negative HER2 status), [ER+|PR+]HER2+ (positive ER, PR and HER2 status), [ER−|PR−]HER2+ (negative ER and PR status, and positive HER2 status), [ER−|PR−]HER2− (negative ER, PR and HER2 status)^8^. Other IHC molecules such as the epidermal growth factor receptor (EGFR) have been identified to classify breast cancers and, in particular, among triple negative tumors^9^,^10^.

Some work focused on the intrinsic breast cancer subgroups using large-scale gene expression profiling with the aid of gene expression array^11^,^12^. Perou *et al*.^13^ identified five subgroups using different gene expression datasets, i.e., Luminal A, Luminal B, HER2, Basal-like tumor, and Normal tumor. The mRNA expression profile of the intrinsic genes was first used by Sørlie *et al*.^12^,^14^ in tumor subgroup identification. Parker *et al*.^15^ developed a classifier, named PAM50, using a 50-gene set to identify the four major intrinsic subtypes. Additionally, microRNAs, a category of small non-coding RNA molecules regulating cell function both at the transcriptional and posttranscriptional levels, complement the prognostic marker discovery using, traditionally, gene expression data^16^,^17^. In this domain, a number of miRNAs, such as miR-7, miR-128a, miR-210^17^, were found differentially expressed among breast cancer subgroups. Dai *et al*.^7^ reported a set of differentially expressed genes (diff-genes), composed of 1015 mRNAs and 69 miRNAs among the four IHC-defined breast tumor subtypes, which was then reduced to a 119 gene panel through a method of feature gene selection^18^.

Exploration on the molecular differences among breast cancer subtypes is of crucial importance in understanding the heterogeneity of breast tumors. Though the “intrinsic” genes can capture the differences among the defined subtypes, they could not tell the pair-wise-subtype differences, which is essential when applied for clinical use. With this aim, our study reveals the significant differences between pair-wise subgroups defined by the major IHC markers (ER, PR and HER2), integrating mRNA and miRNA expression at the transcriptional level. We explore the functional roles of these signature genes and their relationships regarding information flow using network and pathway analysis. In addition, at the transcriptional level, breast cancer subgroups could be identified hierarchically in a pair-wise fashion based on the decision tree, indicating the hierarchical differentiation pattern of breast tumors.

## Results

### Identification of feature genes

**Figure 1:**
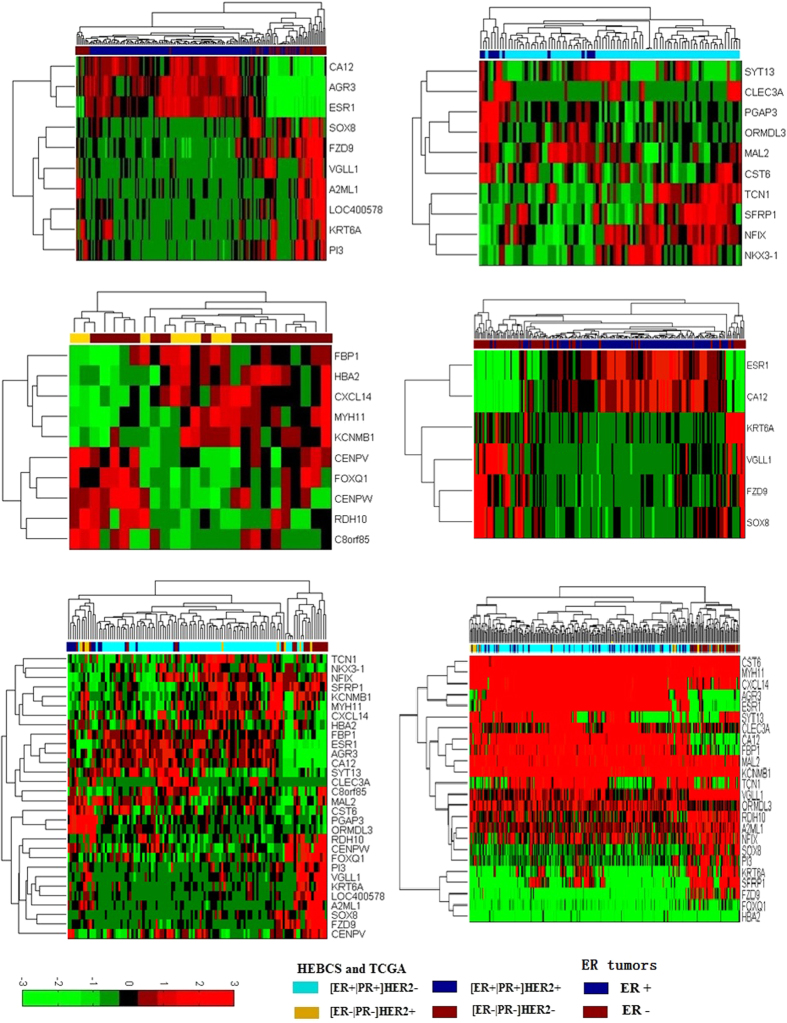
Heatmaps measuring the performance of different gene sets. Using (**A**) mRNA set1 to identify ER+ and ER− tumors in HEBCS; (**B**) mRNA set2 to distinguish [ER+|PR+]HER2+ and [ER+|PR+]HER2− in HEBCS; (**C**) mRNA set3 to identify [ER−|PR−]HER2+ and [ER−|PR−]HER2− in HEBCS; (**D**) gene set 1 to identify ER+ and ER− tumors in GSE22220; (**E**) the feature mRNA sets to classify breast tumors in HEBCS; (**F**) the mRNA feature genes to identify breast tumor subtypes in TCGA.

In HEBCS, out of the 183 invasive tumors, 182 are labeled by the ER status and 115 have the marking information on IHC biomarkers. Four subgroups are defined by ER, PR and HER2, i.e., [ER+|PR+]HER2+, [ER+|PR+]HER2−, [ER−|PR−]HER2+ and [ER−|PR−]HER2−. In detail, the terminologies of different gene sets are listed in Table 1. The mRNA and miRNA feature genes and RSP genes are listed in Supplementary Tables 1 and 2, respectively. The feature gene set contains 30 mRNAs and 8 miRNAs while the RSP gene set is comprised of 31 mRNAs and 19 miRNAs. Worth noting that, no miRNA was found differentially expressed between [ER+|PR+]HER2+ and [ER+|PR+]HER2−. Both hsa-miR-9 and its low-expression form hsa-miR-9* are over-expressed in ER− tumors. Thus, hsa-miR-9* was removed from the final panel to reduce redundancy. In HEBCS, the classifier using gene set1 had a prediction accuracy of 0.8736 (nearest-center classifier) and 0.9066 (naïve Bayesian classifier) in subtypes stratified by ER status; it was 0.8804 (nearest-center classifier) and 0.8804 (naïve Bayesian classifier), respectively, using gene set2 (between subtypes differed by the HER2 status among ER+ tumors), and 0.7692 (nearest-center classifier) and 0.8804 (naïve Bayesian classifier), respectively, using gene set3 (between two subtypes differed by the HER2 status among ER− tumors). These results are shown in Table 2 and Fig. 1A–C. The performance of miRNA and mRNA signature genes was evaluated using different classifiers (SVM: 0.6667 using miRNA genes; 0.7373 with mRNA; 0.7276 integrating mRNA and miRNA genes, Fig. 1E). Tumor patterns identified using the RSP mRNAs and miRNAs were displayed in Supplementary Figs 1 and 2, respectively. Furthermore, pathway analysis with miRNA targets, RSP genes and feature genes was conducted, respectively (Supplementary Table 3). The overlapping pathways suggest the core signaling controlling breast cancer differentiation, such as signaling pathways regulating pluripotency of stem cells and VEGF signaling pathway. MiRNA targets fall into the same pathways with feature mRNAs, indicating the redundancies at these two levels. As miRNAs do not improve the classification accuracy, we include only the 30 mRNAs in the final signature gene panel.

### Validation of feature genes using public datasets

The performance of the feature genes in breast tumor subtyping is validated using GSE22220 and TCGA (Fig. 1D for GSE22220 and Fig. 1F for TCGA). The naïve Bayesian classifiers based on the methods of obtaining the prior knowledge regardless of different platforms were compared with the SVM classifier possessing the training process. The classification accuracies were summarized in Table 2. In GSE22220, the tumors were labeled by ER status, and the classifiers were applied using the mRNA set1. Using the prior knowledge obtained from HEBCS, the nearest-center and naïve Bayesian classifiers achieve an accuracy of 0.7963 and 0.8565, respectively, in GSE22220, and an accuracy of 0.6152 and 0.6242, respectively, in TCGA. Obviously, naïve Bayesian classifier performs better than the nearest-center classifier in identifying tumor subtypes using the feature genes. The SVM classifier comprising of our feature genes is able to differentiate subtypes with an accuracy of 0.8469 and 0.7696, respectively, in GSE22220 and TCGA, which outperforms the other two methods when the training data is available. The results of different classifiers though differ, suggest that the proposed signature gene panel has a good generality. Several classifiers with obtained signature genes perform differently in tumor subtype prediction, with pros and cons summarized in Table 3 and suitable in each specific application.

The feature genes were compared with the RSP genes. Among the 30 mRNA feature genes, 6 overlapped with the RSP feature mRNAs; and out of the 7 miRNA feature genes, 5 overlapped with the 19 RSP miRNAs (Table 4). These overlapping mRNA and miRNA feature genes might be the key molecules driving breast tumor heterogeneity.

### Pathway and network analysis using the signature genes

**Figure 2:**
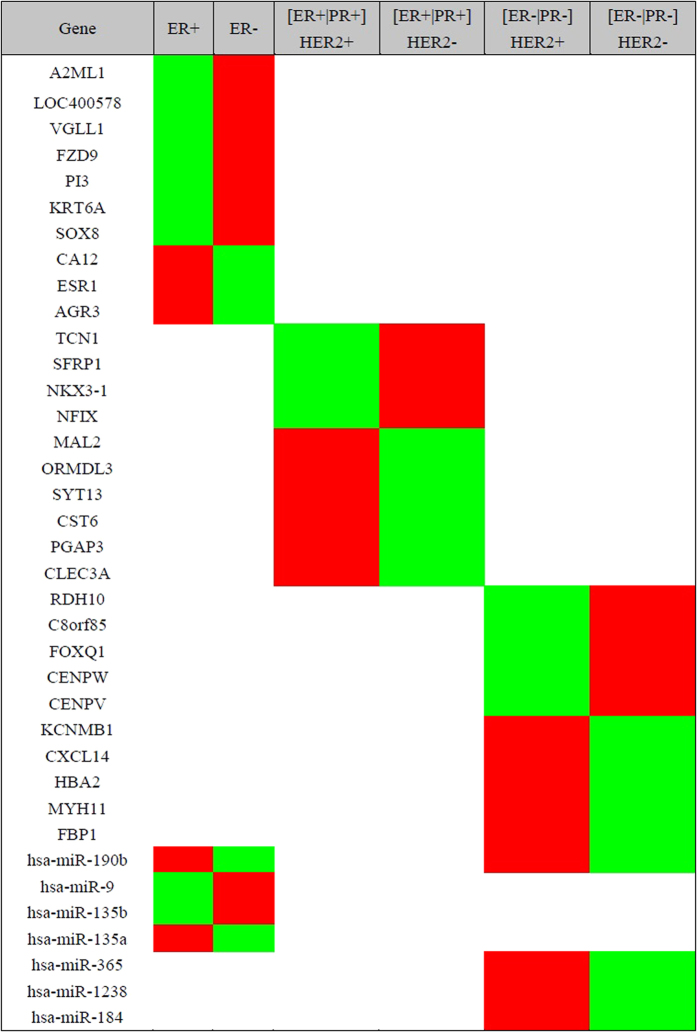
Genes shared in the differentially expressed gene sets among breast cancer subgroups. Genes including mRNA and miRNA, shared among [ER+|PR+]HER2+, [ER+|PR+]HER2−, [ER−|PR−] HER2+ and [ER−|PR−]HER2− subgroups. The over- and under-expression are colored in red and green, respectively, based on the log2-transformed fold change.

The expressions of the feature genes were opposite in a subtype pair-wise fashion, as they are selected based on the differentially expressed degree. These feature genes are rarely shared among different pairs (Fig. 2), suggesting succinctness of the feature genes.

**Figure 3:**
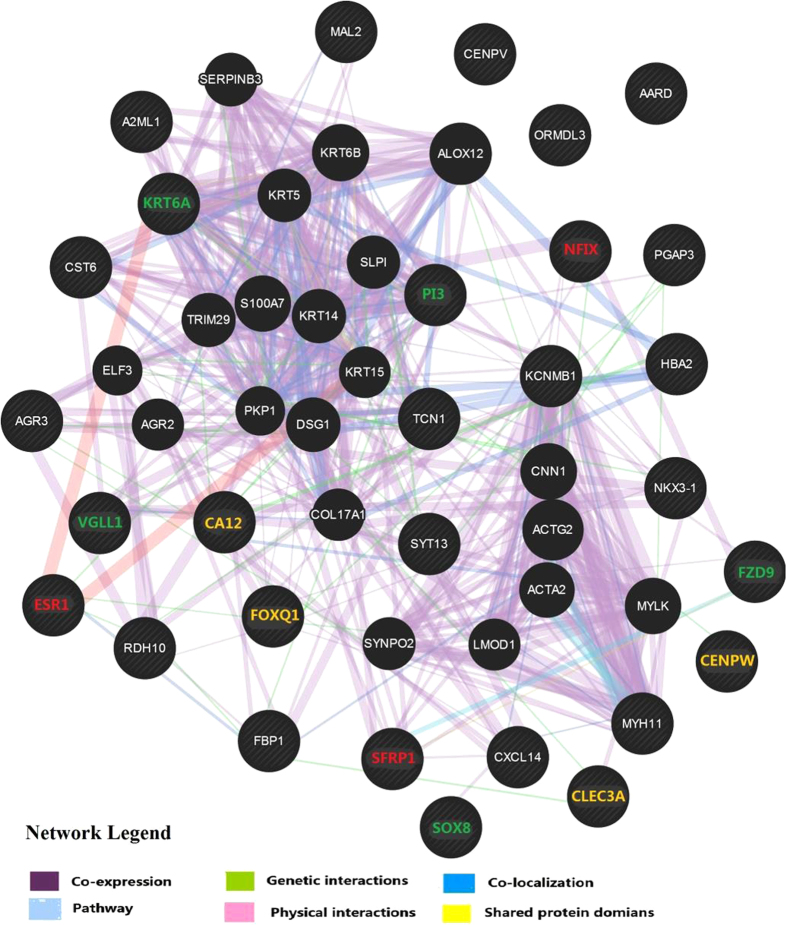
The gene network constructed using GeneMANIA. The network, constructed by the feature genes and 20 related genes, contains 668 links. Different attribution links are labeled by different colors and the mRNA feature genes are indicated with stripes in the gene network.

Several important genes associated with breast cancer, such as ESR1^17^, *FZD9*^19^ and CXCL14^20^, were unveiled. The targets of miRNA feature genes were explored using miRecords. Furthermore, KEGG and KOBAS pathway analysis reveal that several cancer core pathways were enriched in the signature genes, feature genes, as well as their miRNA targets. For example, ESR1^17^, responsive to estrogen related signal and so far the most important molecule distinguishing breast tumor subtypes, is present in the feature set; several well-known molecules associated with Jak-STAT signaling pathway^21^ such as CLEC3A, etc, are enriched in the feature genes and miRNA targets (Supplementary Table 3). FZD9 (from the RSP genes) and several targets of the miRNA feature genes (AKT2, KRAS and NFAT1) are enriched in mTOR (p = 0.037) and VEGF(p = 0.007) signalings^22^. In addition, we checked the diseases relevant to the feature genes, RSP genes and mRNA targets using KEGG disease (Supplementary Table 4), with 56.7% of the enriched diseases being cancers. Gene interaction network was constructed using GeneMANIA according to the physical properties such as co-expression, genetic interaction and pathway. The network of the signature genes contains 49 mRNAs with 20 related genes subjoined and 668 links, among which co-expression attributes 79.5% and co-localization 8% (Fig. 3). In the network constructed using RSP genes (Supplementary Fig. 3), 45 genes in total were connected by 1102 links (co-expression: 81.93%, co-localization: 12.08%, genetic interactions: 4.2% and shared protein domains: 1.79%) with 20 related mRNAs added. Some key genes are densely connected, such as FOXQ1 and SFRP1, which are well-known molecules driving the heterogeneity and progress of breast tumors.

## Discussion

The mRNA and miRNA feature genes, identified using HEBCS, could efficiently differentiate the four IHC-defined tumor subtypes as indicated by the statistics obtained using two other public datasets (Table 2). This suggests that decision tree is an effective approach for identifying feature genes differentiating breast cancer subtypes. We found 6 mRNAs and 5 miRNAs overlapping between the feature genes and RSP genes (Table 3), 3 mRNAs (ESR1, NFIX, SFRP1) overlapping among the signature genes, the Sorlie’s signature and PAM50 genes. Most of the feature genes are shared with Dai’s diff-genes (Table 5), except for C8orf85, CENPW, CENPV, CXCL14, and has-miR-1238, which are revealed only from the decision tree. These overlapping genes may drive breast cancer differentiation, and the 5 genes exceptionally obtained using the decision tree capture the pair-wise differences along the constructed tree assuming that ER is the predominant differentiation factor followed by HER2. With the obtained feature genes, three classifiers are applied to subtype the tumors in the specific usage (Table 4). By using network and pathway analysis, it reveals that co-expression accounts for the most physical properties (79.5% in feature gene network, 81.93% in the RSP gene network), because the gene expression profiling is the key factor to predict the interactions. Some genes, well-known in breast cancer subtyping and involved in the cancer-relevant pathways, are the hubs of the network, e.g., FOXQ1, SFRP1 and ESR1. From the biological view, the roles of selected genes in the regulation of cancer should be analyzed.

### Feature miRNAs

No differentially expressed miRNA was found between the [ER+|PR+]HER2+ and [ER+|PR+]HER2− subgroups, indicating that ER+ tumors are less diverse than ER− tumors. The fact that rather few miRNA was found and less accuracy was obtained once miRNA was included, which might be caused by the following two reasons. First, miRNAs function in driving phenotypic differences through regulating mRNA expression, thus information at these two levels is redundant to some extent. Second, miRNA regulation is a complex and indirect process, with many steps potentially introducing noise, e.g., the feature miRNAs may regulate some non-feature mRNAs, complicating the subtyping process. No overlap was observed between mRNA feature genes and the validated targets of miRNAs, elucidating the concision of the feature genes. Hsa-miR-135a and hsa-miR-135b play crucial roles in distinguishing breast tumors by ER status, which has been extensively discussed in ref. 7. Hsa-miR-9 methylation is reported to be associated with the development of metastasis^23^. In this study, has-miR-9 is over-expressed in ER− tumors, indicating that such an event is crucial in differentiating ER+ and ER− tumors. In MCF-7 docetaxel-resistant breast cancer cells, the miRNA set containing miR-190b was down-regulated^24^. We found hsa-miR-190b be over-expressed in ER+ tumors, and HER2+ tumors among ER− cancers. The fact that hsa-miR-190b is firstly responsive to the hormonal receptor ER and secondarily to the growth receptor HER2, is consistent with the hierarchical structure in our constructed decision tree. This, on one hand, offers the evidence supporting the differentiation hierarchical structure underlying breast cancer heterogeneity and, on the other hand, indicates that the genes under hsa-miR-190b regulation might unveil the key network or pathways driving such a differentiation. We next explored hsa-miR-190b targets, with only predicted ones being found (Supplementary Table 5). The predicted targets of hsa-miR-190b are enriched in some cancer-related pathway (Supplementary Table 5), such as signaling pathways regulating pluripotency of stem cells (p-value = 0.058), cell adhesion molecules (CAMs) (p-value = 0.058)^25^ and AMPK signaling pathway (p-value = 0.042)^26^. Additionally, hsa-miR-365, hsa-miR-1238, hsa-miR-184 are all down-regulated in [ER−|PR−]HER2−, and up-regulated in [ER−|PR−]HER2+. Hsa-miR-365 is over-expressed in human breast cancer which down-regulates IL-6 in HeLa cells^27^. Here we show that its expression could distinguish ER− tumors stratified by HER2 status, and have CXCL14 sharing the same expression pattern with hsa-miR-365, which together suggests the differential regulation of chemokines in breast cancer subtypes. Most of the miRNA targets are known to be involved in cancer-related signalings. For example, AKT2^28^, target of hsa-miR-184, participating in Pten signaling pathway, activates a series of downstream targets, which are involved in the regulation of key cellular functions including cell growth and survival, glucose metabolism and protein translation. KRAS^29^, target of hsa-miR-18a*, has been reported as a genetic marker for development of triple-negative breast cancer in premenopausal women^29^. JAK2^30^, target of hsa-miR-135a, is a key player in JAK-STAT signaling pathway^21^. These three target genes are known to contribute to the regulation of stem cells pluripotency and are related to cancers of the breast and female genital organs. APC, target of has-miR-135b, plays a crucial role in Wnt signaling, and is thus involved in cell-fate specification and progenitor-cell proliferation.

### Feature mRNA set1

Genes belonging to this set stratify breast cancer by ER status. ER, also named ESR1, mediates the biological effects of estrogens through the estrogen response elements (EREs) of the target genes^17^, and has been traditionally applied for breast tumor subtyping and prognosis^3^. As expected, it is found within this set and over-expressed in ER+ tumors. Similarly, CA12 and AGR3 are also up-regulated in ER+ tumors. It is reported that carbonic anhydrase XII (CA12), encoding a zinc metalloenzyme responsible for acidification of the microenvironment of cancer cells, is regulated by estrogen via ERα in breast cancer cells, and that this regulation involves a distal estrogen-responsive enhancer region in human breast tumors^31^. AGR3, also named breast cancer membrane protein 11 (BCMP11), was originally identified as a membrane protein from breast cancer cell lines, which together with AGR2 are both associated with breast cancer and ovarian cancer^32^,^33^.

Also found in this set include A2ML1, LOC400578, VGLL1, FZD9, PI3, KRT6A and SOX8, which are under-expressed in the ER+ group and over-expressed in the ER− group. A2ML1, which encodes the secreted protease inhibitor α-2-macroglobulin (A2M)-like-1, activates mutations in signal transducers of the RAS/mitogen-activated protein kinase (MAPK) pathway^34^. *FZD9*^19^ encodes WNT receptors and is an important factor affecting WNT signaling. MAPK and WNT pathways both contribute in cell proliferation control, suggesting that cell proliferation is a key property driving the differences between ER+ and ER− tumors. Keratin, known as basal markers, also has a member, i.e., KRT6A, found in this gene set. It is reported to have potential relevance to circulating tumor cells, which might function as an early marker for breast cancer metastasis or monitor therapy efficacy^35^. These suggest that metastasis potential is another important index here to differentiate ER+ and ER− tumors. Moreover, several other keratins (e.g., KRT14, KRT15) are included to construct the gene interaction network. VGLL1 (Vestigial-like 1) is a gene encoding a transcriptional co-activator modulating the Hippo pathway, which is known to be associated with a basal-like phenotype in breast cancer^36^. Participation of FZD9 in carcinogenesis has been reported in various cancers, indicating their potential roles in breast cancer, such as mTOR signaling pathway.

### Feature mRNA set2

Genes belonging to this set differentiate ER+ tumors by HER2 status. In particular, TCN1, SFRP1, NKX3-1 and NFIX are suppressed in the [ER+|PR+]HER2+ subtype and up-regulated in the [ER+|PR+]HER2− subtype. TCN1 is reported to be a breast cancer-related gene^37^, which affects replication timing with expression significantly differ between normal and malignant cell lines. SFRP1 encodes the secreted frizzled-related protein 1 which is a soluble Wnt antagonist, and its inactivation is known to be associated with unfavorable prognosis among breast cancer patients^38^. NKX3-1, a prostate-specific tumor suppressor gene is the earliest known marker of prostate epithelium during embryogenesis and is subsequently expressed at all stages of prostate differentiation *in vivo*^39^. Hypermethylated NFIX is identified in the breast cancer model^40^, its differential expression among ER positive tumors as stratified by HER2 status suggests the role of methylation in regulating such a phenotypic difference.

Also found in this set are MAL2, ORMDL3, SYT13, CST6, PGAP3 and CLEC3A, which are lowly expressed in the [ER+|PR+]HER2− subtype but highly expressed in the [ER+|PR+]HER2+ subgroup. MAL2, Mal, T-cell differentiation protein 2, has been identified as a molecule predictive of metastases whose increased expression has been validated in ovarian, colorectal and pancreatic cancer^41^. CST6^42^ is a breast tumor suppressor expressed in normal breast epithelium, but epigenetically silenced as a consequence of promoter hypermethylation in metastatic breast cancer cell lines, which suggests the mechanism of CST6 loss during breast tumorigenesis and/or progression to metastasis. PGAP3 was reported to be specifically expressed in HER2+ tumor cells but not in stroma or HER2 non-amplified breast tumor samples^43^. Its differential expression in [ER+|PR+]HER2− and [ER+|PR+]HER2+ with over-expression in HER2+ luminal tumors is concordant with the previous reports. Synaptotagmin 13 (SYT13) is identified as a putative liver tumor suppressor gene, complementing a molecular defect in GN6TF liver tumor cells and giving rise to tumor suppression through induction of rat WT1^44^. CLEC3A is a heparin-binding, cell adhesion modulator, whose cleavage in tumor microenvironments may affect tumor cell invasion and metastasis by modulating tumor cell adhesion and the plasminogen/plasminogen-activator system^45^. By pathway analysis, CLEC3A participates in signaling pathways regulating pluripotency of stem cells. Genes involved in JAK-STAT signaling are enriched in the gene set 2, with p value = 0.038. Several basic pathways were also involved, such as signaling pathways regulating pluripotency of stem cells and viral carcinogenesis. Variants of ORMDL3 were expressed in human breast cancer cell lines, but the functional relevance of ORMDL3 in breast cancers has not been reported^46^. In our study, it is differentially expressed in ER+ tumors, which provides evidence for their relation.

### Feature mRNA set3

Genes within this set differentiate tumors within ER− tumors as stratified by HER2 status. RDH10, C8orf85, FOXQ1, CENPW and CENPV are suppressed in the [ER−|PR−]HER2+ subgroup, but elevated in the [ER−|PR−]HER2− subgroup. RDH10 was reported to play critical oncogenic roles in tumor progression for patients of non-small-cell lung cancer, and was involved in tumors with lymph node invasion^47^. FOXQ1 expression is regulated by TGF-β1 and involved in the EMT process^48^. Here, its high expression in [ER−|PR−]HER2− tumors as compared with [ER−|PR−]HER2+ accords with our conception that metastasis is an easily gained property of triple negative cancers and suggests its importance in differentiating tumors of these two subtypes. CENPV is required for centromere organization, chromosome alignment and cytokinesis^49^, and CENPW plays crucial roles in the formation of a functional kinetochore involved in cell division during mitosis^50^. Over-expression of these two genes in [ER−|PR−]HER2− tumors suggests the crucial role of irregular cell cycle signaling in triple negative tumors that distinguish it from [ER−|PR−]HER2+ tumors.

KCNMB1, CXCL14, HBA2, MYH11 and FBP1 are over-expressed in [ER−|PR−]HER2+ tumors and under-expressed in [ER−|PR−]HER2− tumors. CXCL14^20^, known as breast and kidney-expressed chemokine (BRAK), is a negative regulator of growth and metastasis, whose expression has a strong association with the overall survival and lymphoid node (LN) metastasis in breast cancer patients. It is up-regulated by reactive oxygen species through the activator protein-1 signaling pathway and promotes cell motility which is verified to be associated with cell adhesion signaling. HBA2 is down-regulated in tumors as compared with normal breast tissue. Its lower expression in [ER−|PR−]HER2− than [ER−|PR−]HER2+ tumors suggests that it is an indicator of cancer stemness and aggressiveness. Recently, MYH1 and MYH9 have been identified as candidate breast cancer genes in a systematic analysis of the breast cancer genome^51^. The differential expression levels of KCNMB1-4 subunits in both MFK223 and MCF7 cell lines leads to differential sensitivity towards physiological agonists (17*β*-estradiol) and pharmacological compounds in breast cancer cell lines^52^. MYH11 plays a role in tumor formation by disturbing stem cell differentiation or affecting cellular energy balance, and has been identified as a driver gene in human colorectal cancer^51^. Its relative low expression in [ER−|PR−]HER2− as compared with [ER−|PR−]HER2+ tumors making the triple negative subtype less differentiated than [ER−|PR−]HER2+ tumors. A retrospective study reported that low FBP1 expression is associated with poor survival^53^, which concords with our observation here that it differentiates [ER−|PR−]HER2− and [ER−|PR−]HER2+ tumors.

## Conclusion

We studied the intrinsic molecular differences of breast cancer subtypes labeled by the three major IHC markers (ER, PR and HER2) in a pair-wise fashion following a decision tree. By presenting a set of feature genes, we capture the differences on molecular profiling among breast cancer subtypes pair-wisely, rather than re-define them into finely grained subgroups. This is fundamentally different from^7^,^18^, where genes differentiating breast tumor subtypes are identified in an ensemble fashion. According to the decision tree constructed using ER, PR and HER2, the feature genes along each branch (gene sets 1 to 3) as well as those differentiating cross-branch pairs (subtype-specific genes) are presented. Gene sets 1 to 3 altogether compose the feature genes. Besides availing in precise diagnosis, genes revealed here could also be utilized to achieve efficient therapeutic treatment of triple negative tumors via modulating the expression of the pivotal genes controlling breast tumor subtype switches. That is, while damoxifen is a commercial drug for luminal A breast cancers, we could apply it for triple negative tumor treatment after applying therapies transiting triple negative tumors into the Luminal A subtype. This could be achieved via modulating the expression of the genes in the corresponding pair. Therefore, these pair-wisely revealed genes have profound clinical implications.

Network and pathway analysis revealed the physical interaction and relationships among the selected feature genes and importance of genes in the gene network and metabolic pathways. Though computational analysis facilitates our understanding towards the functions of these genes, solid experimental validations and functional studies are indispensible to further consolidate our findings before clinical use.

Conclusively, our study bridges the gap between immunohistochemistry markers and gene expression profiling in breast tumor subtyping at the mRNA and miRNA levels, which helps us better understand breast cancer heterogeneity in a pair-wise fashion. More importantly, these genes deepen our understandings towards breast cancer differentiation, and imply an indirect efficient therapeutic strategy for subtypes without targeted therapy.

## Material and Method

### Materials

The three gene expression datasets used in ref. 7 for feature gene identification among breast tumors have been employed in this study. HEBCS was used to identify the differentially expressed gene sets among the IHC-defined subtypes, and GSE22220 together with TCGA was applied to validate the selected gene biomarkers.

HEBCS is comprised of mRNA (GSE24450) and miRNA (GSE43040) expression data and retrieved from the GEO database (Gene Expression Omnibus)^54^, with the experiments carried out at SCIBLU Genomics Centre, Lund University, Sweden. This dataset harbors 24660 mRNAs (Illumina HumanHT-12_V3 Expression BeadChips) and 1104 miRNAs (IlluminaHumanMI_V2 BeadChips) for 183 primary breast tumor samples from the department of Oncology of the Helsinki University Central Hospital (HUCH) and department of Surgery^55^. Among them, 115 tumors were labeled unambiguously by the status of ER, PR and HER2, which were grouped into four subtypes i.e., [ER+|PR+]HER2−, [ER+|PR+]HER2+, [ER−|PR−]HER2+ and [ER−|PR−]HER2−, based on these markers.

GSE22220 is composed of mRNA (GSE22219) and miRNA (GSE22216) expression profiling from GEO^54^. GSE22219 contains 24332 Entrez Gene entities for 216 tumor samples that were processed and hybridized to Illumina Human Ref-8_V1 expression Bead Chips. GSE22216 contains 734 probes (Illumina HumanMI_V1 BeadChips) for 207 samples. Only ER status is available in GSE22220, based on which these samples were grouped into ER+ and ER− tumors.

TCGA dataset (level 3) was retrieved from the TCGA portal at http://tcga.cancer.gov/dataportal, which contains 17814 mRNAs for 451 samples and 1046 miRNAs (IlluminaGA_miRNASeq) for 315 patients. The mRNA dataset was produced from the Agilent 244 K Custom Gene Expression G4502A-07-3 platform, and the miRNA data was generated using IlluminaGA_miRNASeq^7^. These primary solid tumor samples were classified into the four IHC-characterized subtypes as defined in the HEBCS data.

## Methods

### Data normalization

Normalization of gene expression data from different platforms was conducted. The regulatory direction of the listed genes in each set was denoted by*α* ∈ {−1, 0, 1}, with −1, 0, 1 each representing down-, normal- and over- expression, respectively. The average gene expression *x* is denoted by 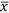, with the standard deviation being marked by δ. The gene expression data was discredited into the status of genes 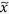, by the following rules:


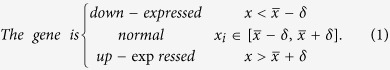


### Decision tree construction and feature gene identification

**Figure 4:**
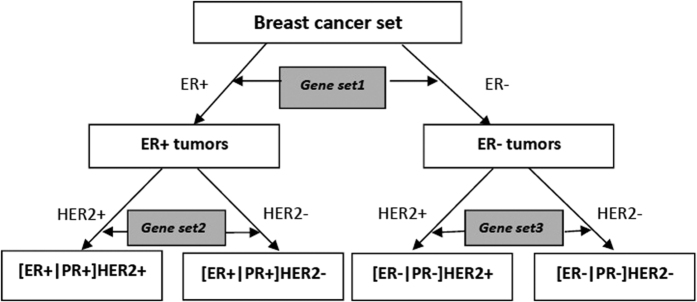
Decision tree for pair-wise identification of the feature genes of breast cancer subtypes. Gene set 1 to 3 (gray) each contains the feature genes identified according to the status of the IHC marker show aside, i.e., ER or HER2. The subtypes are presented as the leaves of the tree.

Breast tumors can be grouped by ER status into ER−positive and ER-negative tumors. Tumors of these two branches could be each further divided into two subtypes by HER2 status, resulting in four subgroups, i.e., [ER+|PR+]HER2+, [ER+|PR+]HER2−, [ER−|PR−]HER2+ and [ER−|PR−]HER2−. Note that PR status is in consistent with that of ER in most cases. This subtype identification procedure can be described by a decision tree (Fig. 4), which splits this complex partitioning process into a union of several simple decisions^56^.

HEBCS data was used to detect the differentially expressed genes which was pre-processed following instructions in ref. 7. Differentially expressed genes were identified in a pair-wise fashion (i.e., ER+ vs. ER−, [ER+|PR+]HER2+ vs. [ER+|PR+]HER2−, and [ER−|PR−]HER2+ vs. [ER−|PR−]HER2−), assuming that ER drives the major difference as indicated by many studies^2^,^57^,^58^. Differentially expressed genes among breast cancer subgroups were selected according to the following rules:

I)  Significant difference on gene expression was observed between pair-wise subtypes under comparison.

II) The average standard deviations in the same subgroup are relatively small.

III) The correlation coefficients of the selected genes are small in absolute value. This is to ensure that the list contains the most succinct number of genes.

The pair-wise identification process is composed of three steps. First, select the distinguishable genes between pair-wise subgroups. That is, genes with base-2 logarithmic fold change larger than 1 as compared with the average expression of each group were chosen. Second, choose differentially expressed genes in a group pair-wise fashion. In this step, the distinguishable genes were filtrated using moderated t-test. That is, the expression level of genes between two subgroups with p-value < 0.05 were considered differentially expressed^59^. Third, measurement of differentially expressed degree was presented. The degree of differential expression was measured by introducing the intra-class difference *MS*_int*ra*_ and inter-class difference *MS*_int*e*r_ which are formulated as 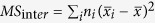 and 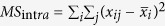, where 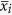 is the average expression of genes in the *i*^th^ group, *n*_*i*_ is the number of the patterns in the *i*^th^ group and 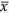 is the average of gene expression. From the statistics point of view^59^,^60^, the bigger *MS*_int*er*_ and the smaller *MS*_int*ra*_ is, the more distinguishable the two groups are. Thus, the index can be defined as


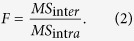


The differentially expressed genes are sorted using the F index. In this study, we considered the difference between two groups, *i* = {1, 2}. If more than 10 genes were differentially expressed, only the top 10 were selected as the feature gene set. Following this process, genes distinguishing ER+ and ER− tumors are called ‘gene set1’, and those discriminating ‘[ER+|PR+]HER2+vs.[ER+|PR+]HER2−’ and ‘[ER−|PR−]HER2+vs.[ER−|PR−] HER2−’ pairs are identified as ‘gene set2’ and ‘gene set3’, respectively. The union of gene sets 1 to 3 is named the ‘feature genes’. Additionally, the differentially expressed genes between the rest-subtype-pairwise genes (RSP genes) were also explored, which contains [ER+|PR+]HER2+ vs. [ER−|PR−]HER2+, [ER+|PR+]HER2+ vs. [ER−|PR−]HER2−,[ER−|PR−]HER2+ vs. [ER+|PR+]HER2− and [ER+|PR+]HER2− vs. [ER−|PR−]HER2− pairs.

### Feature gene validation

A decision tree is constructed to identify the subtypes hierarchically, which can retrieve the prior knowledge from the discovery dataset and apply it to a new dataset based on the normalized gene expression regardless of the experimental platform. The priori probability distribution of feature genes in each subgroup is obtained from the discovery dataset and then applied for subtype identification in a new dataset by the naïve Bayesian classifier using normalized gene expression. Nearest center principle, on the other hand, is a traditional technique for subtype identification and applied here as a comparison. Additionally, as a comparison, SVM classifier is applied to identify the subtypes with kernel functions, equipped with training sets within same platform.

### The naïve Bayesian classifier

The naïve Bayesian classifier^61^,^62^ was applied to calculate the probability that one tumor sample belongs to a certain subgroup. Assuming the signature gene expression is conditionally independent, the conditional probability 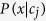 is expressed as 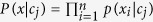, where C is the tumor subtypes and *c*_*j*_ ∈ *C*. We use HEBCS as the discovery data set to train the naïve Bayesian classifier. Given a new pattern with the gene status, the classifier produces a posterior probability distribution over the possible subgroups, i.e.,


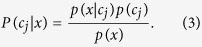


With the goal of assigning tumor samples to the subgroups with the highest accuracy, the objective function is written as


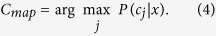


### Nearest center principle

The tumor samples *T*_*x*_ were assigned to the closest group as measured by Euclidean distance, and the nearest-center classifier was designed^63^ as


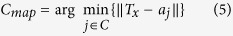


### Network and pathway analysis using feature genes

To investigate the intrinsic heterogeneity of breast cancer, metabolic pathway and network analysis were applied to the obtained feature genes.

**Figure 5:**
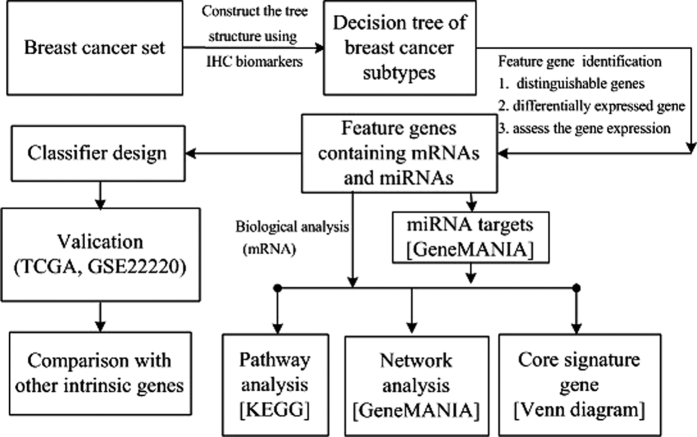
Work flow of the whole process elucidating the differences of breast tumor subtypes. Biological tools used in the analysis are shown in the square brackets.

MiRecords^64^ is a resource for predicting miRNA targets, which integrates experimentally validated miRNA targets having systematic experimental support and predicted miRNA targets using 11 established prediction algorithms (DIANA-microT, MirTarget2 and TargetScan/TargertScanS, etc). It was used to find the targets of the feature miRNA genes. The gene network was constructed using GeneMANIA^65^ (physical attributions: co-expression, co-localization, genetic interactions, pathway, physical interactions, predicted and shared protein domains; automatically selected weighting method was used) to further elucidate the functional roles of the feature genes and the characteristics of each subtype. In addition, we used DAVID^66^ (in functional annotation clustering, similarity term overlap: 3, threshold: 0.5; enrichment thresholds: 1.0 and Benjamini is used. In functional annotation chart, threshold count: 2, ease: 0.1; display way: Benjamini) and KOBAS^67^ (statistical method: hypergeometric test/fisher’s exact test; FDR correction method: Benjamini and Hochberg; Small term cutoff: 5) to interpret the enrichment of gene ontology, metabolic pathways and relevant diseases of these unified feature mRNAs and miRNA targets. The whole process for feature gene identification, validation, and breast tumor heterogeneity exploration, is illustrated in Fig. 5.

## Additional Information

**How to cite this article**: Li, Y. *et al*. Exploring the intrinsic differences among breast tumor subtypes defined using immunohistochemistry markers based on the decision tree. *Sci. Rep.* **6**, 35773; doi: 10.1038/srep35773 (2016).

**Publisher’s note:** Springer Nature remains neutral with regard to jurisdictional claims in published maps and institutional affiliations.

## Supplementary Material

Supplementary Information

## Figures and Tables

**Table 1:** Terminology summary.

Name of set	Description
Gene set1	MRNAs and miRNAs differentially expressed between ER+ and ER− tumors;
Gene set2	MRNAs and miRNAs differentially expressed between [ER+|PR+]HER2+ and [ER+|PR+]HER2−;
Gene set3	MRNAs and miRNAs differentially expressed between [ER−|PR−]HER2+ and [ER−|PR−]HER2−;
mRNA set1 to 3	The mRNAs of gene set 1 to 3;
Feature gene	Unified mRNAs and miRNAs of gene sets 1 to 3;
Signature gene panel	MRNAs of feature gene;
RSP gene(the rest-subtype pairwise genes)	MRNAs and miRNAs differentially expressed in a pair-wise fashion among the rest subtype pairs other than those along the construcgted decision tree. The rest subtype pairs are [ER+|PR+]HER2+ vs. [ER−|PR−]HER2+, [ER+|PR+]HER2+ vs. [ER−|PR−]HER2−, [ER−|PR−]HER2+ vs. [ER+|PR+]HER2−, and [ER+|PR+]HER2− vs. [ER−|PR−]HER2− pairs.

**Table 2:** Comparison of classification accuracy based on the differentially expressed genes using different datasets.

Dataset	Gene	Dimension	Nearest- center classifier	Naïve Bayesian classifier	SVM	Purpose
HEBCS	mRNA set 1	10	0.8736	0.9066	—	Identification
	mRNA set 2	10	0.8804	0.8804	—	Identification
	mRNA set 3	10	0.7692	0.8846	—	Identification
	mRNA feature Genes(signature genes)	30	0.7203	0.7712	0.7373	Validation
	miRNA feature genes	8	—	—	0.6666	Validation
	Feature genes	38	—	—	0.7276	Validation
GSE22220	Gene set 1	6	0.7963	0.8565	0.8469	Validation
TCGA	mRNA feature genes	24	0.6152	0.6242	0.7696	Validation

Note: The gene set 1 to 3, along the decision tree can distinguish the subtypes hierarchically, using nearest center classifier and Naïve Bayesian classifier. The miRNA feature genes are not able to be used for subtyping validation here using neither nearest center classifier nor naïve Bayesian classifier as the hierarchical decision tree was broken with no miRNA found differentially expressed between [ER+|PR+]HER2+ and [ER+|PR+]HER2−. SVM classifier is applied to the miRNA feature genes for subtyping validation using 5-fold cross-validation. Only the mRNA set1 was used for subtyping validation in GSE22220 as only ER status is available in this dataset.

**Table 3:** The comparison of nearest-center classifier, naïve Bayesian classifier and SVM classifier.

Classifier	Advantage	Disadvantage
Nearest-center classifier	Convenient to be applied to the clinical prediction	Linear classifier with unsatisfactory accuracy.
The naïve Bayesian classifier	Obtain the priori probability distribution; Identify the subgroups in same or other datasets.	The prior knowledge obtained based on relatively abundant labeled patterns
SVM classifier	Predict with an acceptable accuracy if training data is available.	Challenging to apply the results to the different datasets

**Table 4:** Overlapping genes between the feature genes and RSP genes.

mRNA			miRNA		
PI3	VGLL1	FZD9	hsa-miR-190b	hsa-miR-184	hsa-miR-135b
LOC400578	KRT6A	SOX8	hsa-miR-135a	hsa-miR-1238	

**Table 5:** The shared genes compared with Dai’s diff-genes.

mRNA	A2ML1	LOC400578	VGLL1	FZD9	ESR1	AGR3
	PI3	KRT6A	SOX8	CA12	TCN1	CST6
	SFRP1	NKX3-1	NFIX	MAL2	SYT13	ORMDL3
	PGAP3	CLEC3A	RDH10	FOXQ1	KCNMB1	HBA2
	MYH11	FBP1				
miRNA	hsa-miR-190b		hsa-miR-9*		hsa-miR-9	
	hsa-miR-135b		hsa-miR-135a		hsa-miR-365	
	hsa-miR-184					
