# Supplementary material for: Exploring the intrinsic differences among breast tumor subtypes defined using immunohistochemistry markers based on the decision tree
Source: Sci Rep. 2016 Oct 27;6:35773. doi: 10.1038/srep35773 (PMC5082366; doi:10.1038/srep35773)
Supplement: Supplementary Information [file srep35773-s1.doc]

**Exploring the intrinsic differences among breast tumor subtypes defined using immunohistochemistry markers based on the decision tree**

Yang Li^1^, Xu_Qing Tang^1^, Zhonghu Bai^2,3^, Xiaofeng Dai^*2,3^ ,

1: School of Science, Jiangnan University, Wuxi 214122, China

2: National Engineering Laboratory for Cereal Fermentation Technology, Jiangnan University, Wuxi 214122, China

3: School of Biotechnology, Jiangnan University, Wuxi 214122, China

Corresponding author:

Xiaofeng Dai

Email: xiaofeng.dai@me.com

**Supplementary Information**

This file contains all the supplementary figures and tables, as listed below.

**Supplementary Table 1. The differentially expressed mRNAs between the pairwise breast cancer subtypes defined by the IHC biomarker information**

**Supplementary Table 2. The differentially expressed miRNAs between the pair-wise breast cancer subtypes defined by the IHC biomarker information**

**Supplementary Table 3. Pathways analysis of the feature genes, RSP genes and miRNA targets**

**Supplementary Table 4. KEGG disease analysis of the feature genes, RSP genes and miRNA targets**

**Supplementary Table 5. Targets of has-miR-190b**

**Supplementary Table 6. Pathway analysis of targets of has-miR-190b**

**Supplementary Figure 1**. **Tumors defined by the IHC molecules from HEBCS identified using the signature genes on basis of mRNA expression.** (A) [ER+|PR+]HER2+ vs. [ER-|PR-]HER2+; (B) [ER+|PR+]HER2+ vs. [ER-|PR-]HER2-; (C) [ER+|PR+]HER2- vs. [ER-|PR-]HER2+ (D) [ER-|PR-]HER2+ vs. [ER-|PR-]HER2-.

**Supplementary Figure 2**. **Tumors defined by the IHC molecules identified on basis of miRNA expression.** (A) ER+ vs. ER- tumors; (B) [ER+|PR+]HER2+ vs. [ER-|PR-]HER2+; (C) [ER+|PR+]HER2+ vs. [ER-|PR-]HER2- (D) [ER+|PR+]HER2- vs. [ER-|PR-]HER2+, (E) [ER+|PR+]HER2- vs. [ER-|PR-]HER2-, (F) [ER-|PR-]HER2+ vs. [ER-|PR-]HER2-

**Supplementary Figure 3**. **The gene interaction network is constructed by using GeneMANIA.**  47 total genes (27 indicated and 20 related) and 1111 links are contained. Different interaction attributions including co-expression, co-localization, genetic interaction and shared protein domain, are marked by different colors and the searched genes are addressed by the stripes.

**Supplementary tables**

**Supplementary Table 1. The differentially expressed mRNAs between the pair-wise breast cancer subtypes defined by the IHC biomarker information**

| **Pairwise subtypes** | **Pairwise feature mRNA** |
| --- | --- |
| Gene set1  (ER+ VS. ER-) | A2ML1, LOC400578, VGLL1, FZD9, PI3, KRT6A, CA12, ESR1, AGR3, SOX8; |
| Gene set 2  ([ER+|PR+]HER2+ VS. [ER+|PR+]HER2-) | TCN1, MAL2, SFRP1, ORMDL3, NKX3-1, SYT13, CST6, NFIX, PGAP3, CLEC3A |
| Gene set 3  ([ER-|PR-]HER2+ VS. [ER-|PR-]HER2-) | RDH10, C8orf85, FOXQ1, CENPW, CENPV, KCNMB1, CXCL14, HBA2, MYH11, FBP1 |
| [ER+|PR+]HER2+ VS. [ER-|PR-]HER2+ | STAC2, FAM5C, HPD, PROL1, PGC, SOX10, TRIM29, SOX8, VGLL1, ROPN1 |
| [ER+|PR+]HER2+ VS. [ER-|PR-]HER2- | ROPN1, SOX8, SOX10, FAM3D, PI15, CSN3, LEMD1, VGLL1, FZD9, ART3 |
| [ER-|PR-]HER2+ VS. [ER+|PR+]HER2- | NAT1, PGR, LOC647654, C6orf211, RTN1 GFRA1, TMEM145, RPL7P9, HDGFRP3, RPL7 |
| [ER+|PR+]HER2- VS. [ER-|PR-]HER2- | TTYH1, LOC400578, VGLL1, CSN3, FZD9, KRT6A, SOX8, FAM3D, PI3, ART3 |

**Supplementary Table 2. The differentially expressed miRNAs between the pair-wise breast cancer subtypes defined by the IHC biomarker information**

| **Pairwise subtypes** | **Pairwise feature miRNA** |
| --- | --- |
| Gene set1  (ER+ VS. ER-) | hsa-miR-190b, hsa-miR-9*, hsa-miR-135b, hsa-miR-9, hsa-miR-135a |
| Gene set 2  ([ER+|PR+]HER2+ VS. [ER+|PR+]HER2-) |  |
| Gene set 3  ([ER-|PR-]HER2+ VS. [ER-|PR-]HER2-) | hsa-miR-365, hsa-miR-190b, hsa-miR-1238, hsa-miR-184 |
| [ER+|PR+]HER2+ VS. [ER-|PR-]HER2+ | hsa-miR-184, hsa-miR-135b, hsa-miR-1238, hsa-miR-142-5p |
| [ER+|PR+]HER2+ VS. [ER-|PR-]HER2- | hsa-miR-577, hsa-miR-135b, hsa-miR-518e*, hsa-miR-190b, hsa-miR-149, hsa-miR-33b, hsa-miR-449a |
| [ER-|PR-]HER2+ VS. [ER+|PR+]HER2- | hsa-miR-181c*,hsa-miR-224, hsa-miR-187, hsa-miR-452, hsa-miR-190b, hsa-miR-1290,hsa-miR-184, hsa-miR-1238, hsa-miR-135a |
| [ER+|PR+]HER2- VS. [ER-|PR-]HER2- | hsa-miR-577, hsa-miR-518e*, hsa-miR-190b, hsa-miR-522, hsa-miR-18a*, hsa-miR-452, hsa-miR-135b, hsa-miR-9*, hsa-miR-149, hsa-miR-224 |

**Supplementary Table 3. Pathways analysis of the feature genes, RSP genes and miRNA targets**

| **ID** | **Term** | **Feature gene** | **P-value1** | **RSP gene** | **P-value2** | **MiRNA targets** | **P-value3** |
| --- | --- | --- | --- | --- | --- | --- | --- |
| hsa00120 | Primary bile acid biosynthesis | CENPW | 0.01 |  |  |  |  |
| hsa00140 | Steroid hormone biosynthesis | CENPV | 0.02 |  |  |  |  |
| hsa00565 | Ether lipid metabolism | ESR1 | 0.07 |  |  |  |  |
| hsa00590 | Arachidonic acid metabolism | ESR1 | 0.09 |  |  |  |  |
| hsa00591 | Linoleic acid metabolism | ESR1 | 0.04 |  |  |  |  |
| hsa00592 | alpha-Linolenic acid metabolism | ESR1 | 0.04 |  |  |  |  |
| hsa00970 | Aminoacyl-tRNA biosynthesis | MYH11 | 0.02 |  |  |  |  |
| hsa01100 | Metabolic pathways | CENPV, CENPW | 0.06 |  |  |  |  |
| hsa04060 | Cytokine-cytokine receptor interaction | CLEC3A | 0.06 |  |  |  |  |
| hsa04146 | Peroxisome | CENPW | 0.03 |  |  |  |  |
| hsa04150 | mTOR signaling pathway | FZD9 | 0.09 | FZD9 | 0.04 |  |  |
| hsa04370 | VEGF signaling pathway | ESR1 | 0.09 |  |  | AKT2,KRAS,NFAT1 | 0.01 |
| hsa04550 | Signaling pathways regulating pluripotency of stem cells | CLEC3A | 0.03 |  |  | AKT2,JAK2,KRAS | 0.06 |
| hsa04610 | Complement and coagulation cascades | HBA2 | 0.02 |  |  |  |  |
| hsa04630 | Jak-STAT signaling pathway | CLEC3A | 0.04 |  |  |  |  |
| hsa04666 | Fc gamma R-mediated phagocytosis | FZD9, ESR1 | 0.02 | FZD9 | 0.06 |  |  |
| hsa04730 | Long-term depression | ESR1 | 0.09 |  |  |  |  |
| hsa04913 | Ovarian steroidogenesis | CENPV, ESR1 | 0.01 |  |  |  |  |
| hsa05010 | Alzheimers disease | CST6 | 0.04 |  |  |  |  |
| hsa05033 | Nicotine addiction | CA12 | 0.06 |  |  |  |  |
| hsa05144 | Malaria | CST6 | 0.01 |  |  |  |  |
| hsa05203 | Viral carcinogenesis | CLEC3A | 0.05 |  |  |  |  |
| hsa05221 | Acute myeloid leukemia | FZD9 | 0.08 | FZD9 | 0.04 | AKT2,KRAS | 0.05 |
| hsa05231 | Choline metabolism in cancer | FZD9, ESR1 | 0.02 | FZD9 | 0.06 |  |  |
| hsa03008 | Ribosome biogenesis in eukaryotes |  |  | TTYH1 | 0.05 |  |  |
| hsa03010 | Ribosome |  |  | GABRP | 0.08 |  |  |
| hsa04012 | ErbB signaling pathway |  |  | FZD9 | 0.05 |  |  |
| hsa04066 | HIF-1 signaling pathway |  |  | FZD9 | 0.06 |  |  |
| hsa04152 | AMPK signaling pathway |  |  | FZD9 | 0.07 |  |  |
| hsa04350 | TGF-beta signaling pathway |  |  | FZD9 | 0.05 |  |  |
| hsa04520 | Adherens junction |  |  | CSN3 | 0.04 |  |  |
| hsa04910 | Insulin signaling pathway |  |  | FZD9 | 0.08 |  |  |
| hsa05014 | Amyotrophic lateral sclerosis (ALS) |  |  | FAM3D | 0.03 |  |  |
| hsa04660 | T cell receptor signaling pathway |  |  |  |  | AKT2,KRAS,NFAT1 | 0.03 |
| hsa04662 | B cell receptor signaling pathway |  |  |  |  | AKT2,KRAS,NFAT1 | 0.01 |
| hsa04664 | Fc epsilon RI signaling pathway |  |  |  |  | AKT2,KRAS | 0.07 |
| hsa04725 | Cholinergic synapse |  |  |  |  | AKT2,JAK2,KRAS | 0.03 |
| hsa04917 | Prolactin signaling pathway |  |  |  |  | AKT2,JAK2,KRAS | 0.01 |
| hsa04920 | Adipocytokine signaling pathway |  |  |  |  | AKT2,JAK2 | 0.07 |
| hsa05161 | Hepatitis B |  |  |  |  | AKT2,KRAS,NFAT1 | 0.06 |
| hsa05210 | Colorectal cancer |  |  |  |  | AKT2,KRAS | 0.06 |
| hsa05211 | Renal cell carcinoma |  |  |  |  | AKT2,KRAS | 0.06 |
| hsa05212 | Pancreatic cancer |  |  |  |  | AKT2,KRAS | 0.06 |
| hsa05213 | Endometrial cancer |  |  |  |  | AKT2,KRAS | 0.04 |
| hsa05214 | Glioma |  |  |  |  | AKT2,KRAS | 0.06 |
| hsa05218 | Melanoma |  |  |  |  | AKT2,KRAS | 0.07 |
| hsa05220 | Chronic myeloid leukemia |  |  |  |  | AKT2,KRAS | 0.08 |
| hsa05223 | Non-small cell lung cancer |  |  |  |  | AKT2,KRAS | 0.05 |
| hsa05230 | Central carbon metabolism in cancer |  |  |  |  | AKT2,KRAS | 0.07 |

**Supplementary Table 4. KEGG disease analysis of the feature genes, RSP genes and miRNA targets**

| **ID** | **Term** | **Feature mRNA** | **P-value1** | **RSP mRNA** | **P-value2** | **miRNA target** | **P-value3** |
| --- | --- | --- | --- | --- | --- | --- | --- |
| H00407 | Peroxisomal beta-oxidation enzyme deficiency | CENPW | 0.01 |  |  |  |  |
| H00476 | Multiple epiphyseal dysplasia (MED) | CXCL14 | 0.01 |  |  |  |  |
|  | Peroxisomal diseases | CENPW | 0.02 |  |  |  |  |
|  | Congenital disorders of lipid/glycolipid metabolism | CENPW | 0.07 |  |  |  |  |
|  | Peroxisomal diseases | CENPW | 0.02 |  |  |  |  |
|  | Congenital disorders of lipid/glycolipid metabolism | CENPW | 0.07 |  |  |  |  |
| H00101 | Other phagocyte defects |  |  | ART3 | 0.01 |  |  |
| H00058 | Amyotrophic lateral sclerosis (ALS) |  |  | FAM3D | 0.01 |  |  |
|  | Primary immunodeficiency |  |  | ART3 | 0.10 |  |  |
| H00027 | Ovarian cancer |  |  |  |  | AKT2,KRAS | 0.00 |
|  | Cancers of the breast and female genital organs |  |  |  |  | AKT2,KRAS | 0.01 |
|  | Cancers of haematopoietic and lymphoid tissues |  |  |  |  | KRAS,JAK2 | 0.02 |
| H00041 | Kaposi's sarcoma |  |  |  |  | KRAS | 0.03 |
| H00046 | Cholangiocarcinoma |  |  |  |  | KRAS | 0.03 |
| H00026 | Endometrial cancer |  |  |  |  | KRAS | 0.03 |
| H00019 | Pancreatic cancer |  |  |  |  | KRAS | 0.04 |
| H00030 | Cervical cancer |  |  |  |  | KRAS | 0.04 |
| H00016 | Oral cancer |  |  |  |  | KRAS | 0.04 |
| H00010 | Multiple myeloma |  |  |  |  | KRAS | 0.05 |
|  | Cancers |  |  |  |  | AKT2,KRAS,JAK2 | 0.05 |
| H00458 | Craniosynostosis |  |  |  |  | KRAS | 0.05 |
| H00014 | Non-small cell lung cancer |  |  |  |  | KRAS | 0.06 |
| H00003 | Acute myeloid leukemia (AML) |  |  |  |  | KRAS | 0.06 |
| H00048 | Hepatocellular carcinoma |  |  |  |  | KRAS | 0.06 |
|  | Skin cancers |  |  |  |  | KRAS | 0.06 |
| H00020 | Colorectal cancer |  |  |  |  | KRAS | 0.07 |
| H00523 | Noonan syndrome |  |  |  |  | KRAS | 0.07 |
|  | Head and neck cancers |  |  |  |  | KRAS | 0.07 |
| H00032 | Thyroid cancer |  |  |  |  | KRAS | 0.08 |
| H00018 | Gastric cancer |  |  |  |  | KRAS | 0.10 |

**Supplementary Table 5. Targets of has-miR-190b**

| **Gene symbol** | **Description** |
| --- | --- |
| NBEA | neurobeachin |
| ACTG1 | actin, gamma 1 |
| AGK | acylglycerol kinase |
| AMACR | alpha-methylacyl-CoA racemase |
| ANGPTL1 | angiopoietin-like 1 |
| AP1S2 | adaptor-related protein complex 1, sigma 2 subunit |
| AP1S3 | adaptor-related protein complex 1, sigma 3 subunit |
| ARHGEF12 | Rho guanine nucleotide exchange factor (GEF) 12 |
| BBS4 | Bardet-Biedl syndrome 4 |
| BCKDHB | branched chain keto acid dehydrogenase E1, beta polypeptide (maple syrup urine disease) |
| BCL11A | B-cell CLL/lymphoma 11A (zinc finger protein) |
| BRUNOL4 | bruno-like 4, RNA binding protein (Drosophila) |
| C5orf33 | chromosome 5 open reading frame 33 |
| CALCR | calcitonin receptor |
| CASP2 | caspase 2, apoptosis-related cysteine peptidase (neural precursor cell expressed, developmentally down-regulated 2) |
| CHD7 | chromodomain helicase DNA binding protein 7 |
| CKAP2 | cytoskeleton associated protein 2 |
| CLEC12B | C-type lectin domain family 12, member B |
| CNN3 | calponin 3, acidic |
| CPOX | coproporphyrinogen oxidase |
| CRISPLD2 | cysteine-rich secretory protein LCCL domain containing 2 |
| CROT | carnitine O-octanoyltransferase |
| CSN2 | casein beta |
| CYP2U1 | cytochrome P450, family 2, subfamily U, polypeptide 1 |
| DAG1 | dystroglycan 1 (dystrophin-associated glycoprotein 1) |
| DDEF1 | development and differentiation enhancing factor 1 |
| DDEF2 | development and differentiation enhancing factor 2 |
| DHRS12 | dehydrogenase/reductase (SDR family) member 12 |
| DMD | dystrophin (muscular dystrophy, Duchenne and Becker types) |
| DNAJB14 | DnaJ (Hsp40) homolog, subfamily B, member 14 |
| DOCK9 | dedicator of cytokinesis 9 |
| EFR3A | EFR3 homolog A (S. cerevisiae) |
| EMR3 | egf-like module containing, mucin-like, hormone receptor-like 3 |
| EPB41L4B | erythrocyte membrane protein band 4.1 like 4B |
| ERG | v-ets erythroblastosis virus E26 oncogene homolog (avian) |
| F2R | coagulation factor II (thrombin) receptor |
| FAM122A | family with sequence similarity 122A |
| FLJ20160 | FLJ20160 protein |
| FNDC3A | fibronectin type III domain containing 3A |
| FNIP1 | folliculin interacting protein 1 |
| FOXJ3 | forkhead box J3 |
| GCLC | glutamate-cysteine ligase, catalytic subunit |
| GPHN | gephyrin |
| GRAMD1C | GRAM domain containing 1C |
| GYG1 | glycogenin 1 |
| HBS1L | HBS1-like (S. cerevisiae) |
| HCN1 | hyperpolarization activated cyclic nucleotide-gated potassium channel 1 |
| HECA | headcase homolog (Drosophila) |
| HIATL1 | hippocampus abundant transcript-like 1 |
| IKZF2 | IKAROS family zinc finger 2 (Helios) |
| ISOC1 | isochorismatase domain containing 1 |
| KBTBD7 | kelch repeat and BTB (POZ) domain containing 7 |
| KCTD12 | potassium channel tetramerisation domain containing 12 |
| KCTD3 | potassium channel tetramerisation domain containing 3 |
| KIAA1033 | KIAA1033 |
| KIAA2018 | KIAA2018 |
| KLF15 | Kruppel-like factor 15 |
| KLHDC5 | kelch domain containing 5 |
| KLHL7 | kelch-like 7 (Drosophila) |
| LMBRD2 | LMBR1 domain containing 2 |
| LNX2 | ligand of numb-protein X 2 |
| LOC153364 | similar to metallo-beta-lactamase superfamily protein |
| LRP11 | low density lipoprotein receptor-related protein 11 |
| LRRC31 | leucine rich repeat containing 31 |
| LRRTM2 | leucine rich repeat transmembrane neuronal 2 |
| MED4 | mediator complex subunit 4 |
| MEGF10 | multiple EGF-like-domains 10 |
| MMAA | methylmalonic aciduria (cobalamin deficiency) cblA type |
| MPZL1 | myelin protein zero-like 1 |
| MUC17 | mucin 17, cell surface associated |
| MYCBP2 | MYC binding protein 2 |
| MYEF2 | myelin expression factor 2 |
| MYH11 | myosin, heavy chain 11, smooth muscle |
| MYO5A | myosin VA (heavy chain 12, myoxin) |
| NAV3 | neuron navigator 3 |
| NCOA7 | nuclear receptor coactivator 7 |
| NDFIP2 | Nedd4 family interacting protein 2 |
| NEGR1 | neuronal growth regulator 1 |
| NEUROD1 | neurogenic differentiation 1 |
| NLGN1 | neuroligin 1 |
| ORC4L | origin recognition complex, subunit 4-like (yeast) |
| OTUD4 | OTU domain containing 4 |
| PAX3 | paired box 3 |
| PAX6 | paired box 6 |
| PCDH17 | protocadherin 17 |
| PCDH9 | protocadherin 9 |
| PDE7B | phosphodiesterase 7B |
| PFDN4 | prefoldin subunit 4 |
| PHF20L1 | PHD finger protein 20-like 1 |
| POF1B | premature ovarian failure, 1B |
| PPP2R2C | protein phosphatase 2 (formerly 2A), regulatory subunit B, gamma isoform |
| PPP2R5C | protein phosphatase 2, regulatory subunit B", gamma isoform |
| RAB8A | RAB8A, member RAS oncogene family |
| RBAK | RB-associated KRAB zinc finger |
| RFK | riboflavin kinase |
| RGL1 | ral guanine nucleotide dissociation stimulator-like 1 |
| RNF12 | ring finger protein 12 |
| RNF125 | ring finger protein 125 |
| RNF144B | ring finger 144B |
| RPS6KA3 | ribosomal protein S6 kinase, 90kDa, polypeptide 3 |
| SEC23A | Sec23 homolog A (S. cerevisiae) |
| SFRS2IP | splicing factor, arginine/serine-rich 2, interacting protein |
| SLC2A2 | solute carrier family 2 (facilitated glucose transporter), member 2 |
| SMAD2 | SMAD family member 2 |
| STK17A | serine/threonine kinase 17a |
| STS | steroid sulfatase (microsomal), isozyme S |
| TBC1D14 | TBC1 domain family, member 14 |
| TCF4 | transcription factor 4 |
| TEX15 | testis expressed 15 |
| TMEM161B | transmembrane protein 161B |
| TMEM45B | transmembrane protein 45B |
| TNRC6A | trinucleotide repeat containing 6A |
| TNRC6B | trinucleotide repeat containing 6B |
| TRIM33 | tripartite motif-containing 33 |
| TRIM36 | tripartite motif-containing 36 |
| TRIM55 | tripartite motif-containing 55 |
| TRPS1 | trichorhinophalangeal syndrome I |
| WDFY3 | WD repeat and FYVE domain containing 3 |
| WSB1 | WD repeat and SOCS box-containing 1 |
| XRN1 | 5"-3" exoribonuclease 1 |
| YTHDF3 | YTH domain family, member 3 |
| ZADH2 | zinc binding alcohol dehydrogenase domain containing 2 |
| ZBTB11 | zinc finger and BTB domain containing 11 |
| ZFP1 | zinc finger protein 1 homolog (mouse) |
| ZIC3 | Zic family member 3 heterotaxy 1 (odd-paired homolog, Drosophila) |
| ZNF229 | zinc finger protein 229 |
| ZNF323 | zinc finger protein 323 |
| ZNF665 | zinc finger protein 665 |

**Supplementary Table 6. Pathway analysis of targets of has-miR-190b**

| **ID** | **term** | **gene** | **p-value** |
| --- | --- | --- | --- |
| hsa04950 | Maturity onset diabetes of the young | NEUROD1,PAX6,SLC2A2 | 0.000704 |
| hsa04144 | Endocytosis | KIAA1033,SMAD2,RAB8A,DDEF2,F2R,DDEF1 | 0.005477 |
| hsa05416 | Viral myocarditis | DMD,DAG1,ACTG1 | 0.006026 |
| hsa05412 | Arrhythmogenic right ventricular cardiomyopathy (ARVC) | DMD,DAG1,ACTG1 | 0.01142 |
| hsa05410 | Hypertrophic cardiomyopathy (HCM) | DMD,DAG1,ACTG1 | 0.015363 |
| hsa05414 | Dilated cardiomyopathy | DMD,DAG1,ACTG1 | 0.018367 |
| hsa03015 | mRNA surveillance pathway | PPP2R2C,HBS1L,PPP2R5C | 0.019435 |
| hsa04152 | AMPK signaling pathway | PPP2R2C,RAB8A,PPP2R5C | 0.041918 |
| hsa04611 | Platelet activation | ACTG1,F2R,ARHGEF12 | 0.046971 |
| hsa04530 | Tight junction | MYH11,PPP2R2C,ACTG1 | 0.054153 |
| hsa04550 | Signaling pathways regulating pluripotency of stem cells | ZIC3,PAX6,SMAD2 | 0.05793 |
| hsa04514 | Cell adhesion molecules (CAMs) | NEGR1,MPZL1,NLGN1 | 0.05793 |
| hsa04390 | Hippo signaling pathway | PPP2R2C,ACTG1,SMAD2 | 0.069982 |
| hsa04917 | Prolactin signaling pathway | CSN2,SLC2A2 | 0.07391 |
| hsa04520 | Adherens junction | ACTG1,SMAD2 | 0.075644 |
| hsa04146 | Peroxisome | CROT,AMACR | 0.093655 |

**Supplementary figures**


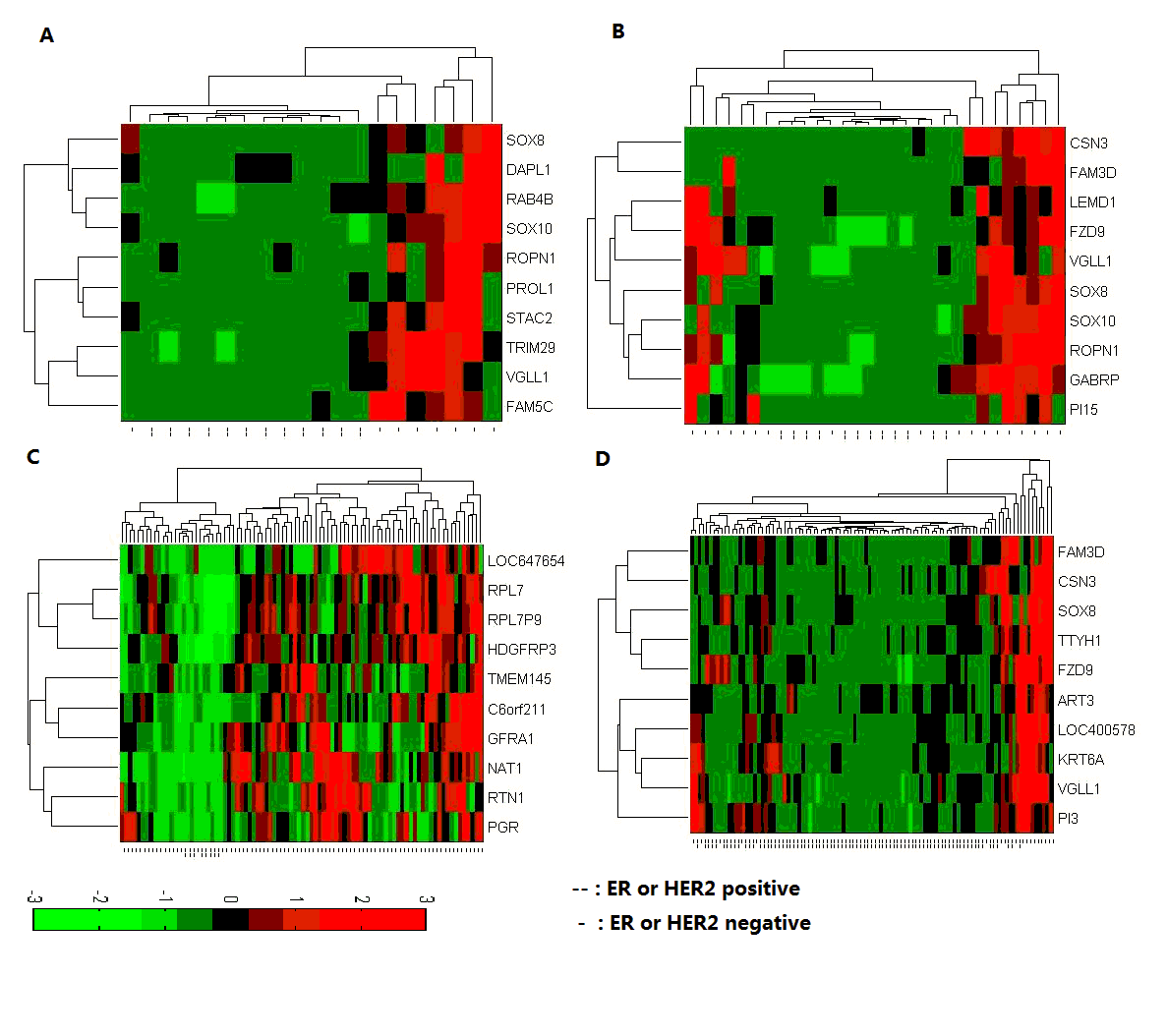


**Supplementary Figure 1**. **Tumors defined by the IHC molecules from HEBCS identified using the signature genes on basis of mRNA expression.** (A) [ER+|PR+]HER2+ vs. [ER-|PR-]HER2+; (B) [ER+|PR+]HER2+ vs. [ER-|PR-]HER2-; (C) [ER+|PR+]HER2- vs. [ER-|PR-]HER2+ (D) [ER-|PR-]HER2+ vs. [ER-|PR-]HER2-.


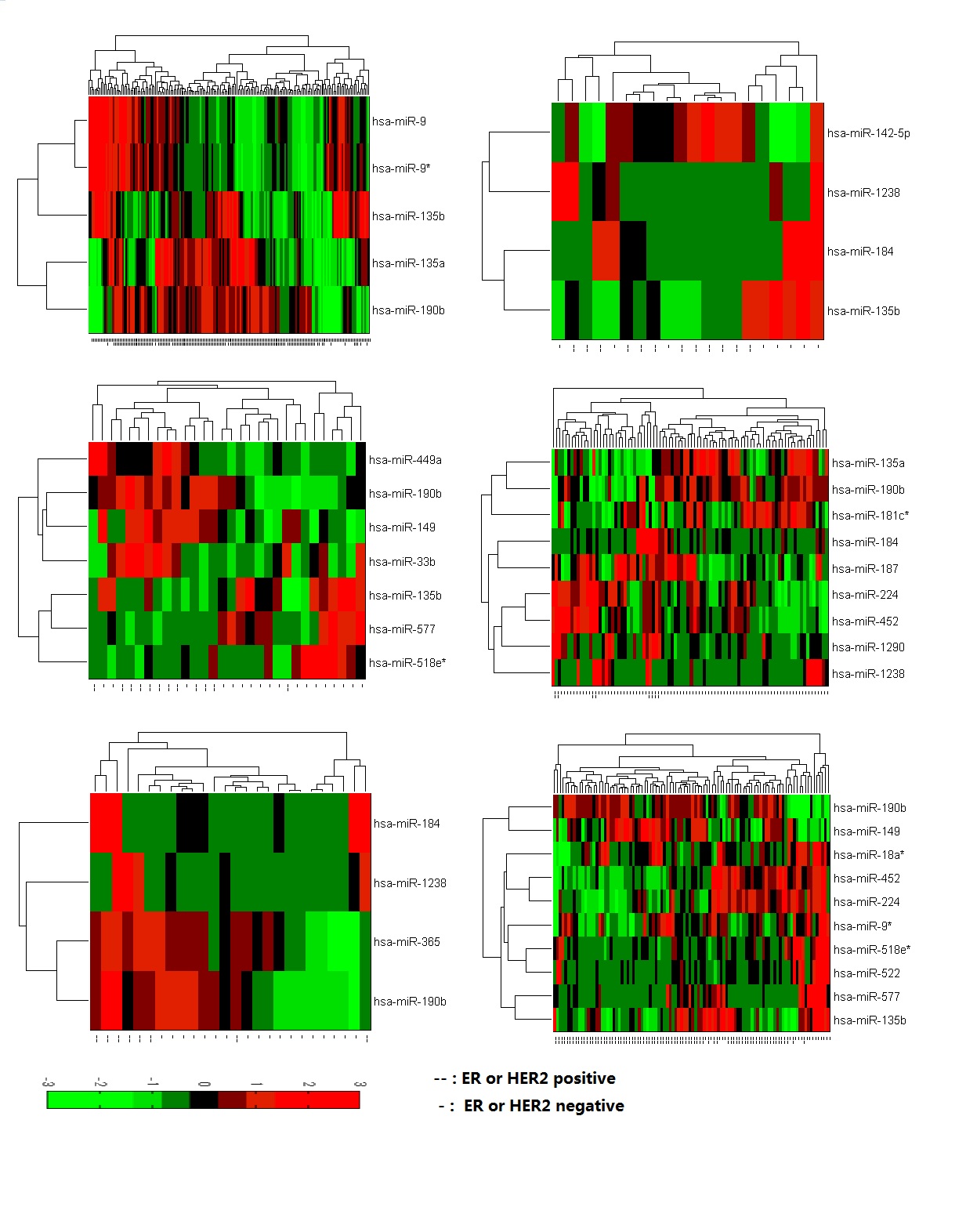


**Supplementary Figure 2**. **Tumors defined by the IHC molecules identified on basis of miRNA expression.** (A) ER+ vs. ER- tumors; (B) [ER+|PR+]HER2+ vs. [ER-|PR-]HER2+; (C) [ER+|PR+]HER2+ vs. [ER-|PR-]HER2- (D) [ER+|PR+]HER2- vs. [ER-|PR-]HER2+, (E) [ER+|PR+]HER2- vs. [ER-|PR-]HER2-, (F) [ER-|PR-]HER2+ vs. [ER-|PR-]HER2-


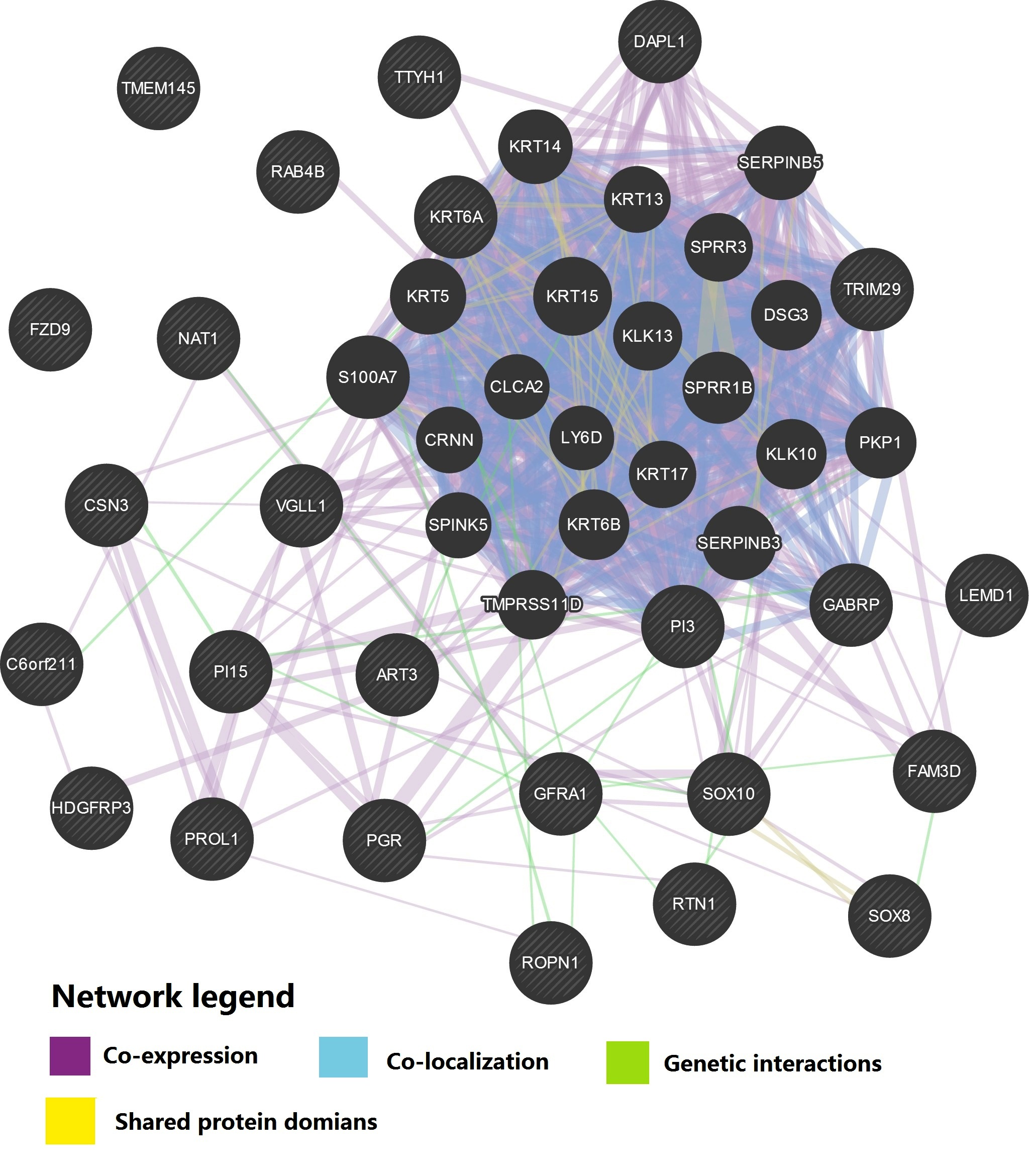


**Supplementary Figure 3**. **The gene interaction network is constructed by using GeneMANIA.**  47 total genes (27 indicated and 20 related) and 1111 links are contained. Different interaction attributions including co-expression, co-localization, genetic interaction and shared protein domain, are marked by different colors and the searched genes are addressed by the stripes.
